# Supplementary material for: Globular pattern formation of hierarchical ceria nanoarchitectures
Source: Commun Chem. 2024 Jun 12;7:128. doi: 10.1038/s42004-024-01199-y (PMC11549420; doi:10.1038/s42004-024-01199-y)
Supplement: Supplementary file 2 — Supplementary Information [file 42004_2024_1199_MOESM2_ESM.pdf]

Supplementary Information

Globular pattern formation of hierarchical ceria nanoarchitectures

Noboru Aoyagi[1*], Ryuhei Motokawa[1,2], Masahiko Okumura[3], Yuki, Ueda[2], Takumi Saito[1,4], Shotaro Nishitsuji[5], Tomitsugu Taguchi[6], Takumi Yomogida[7], Gen Sazaki[8] and Atsushi Ikeda-Ohno[1]

[1]Advanced Science Research Centre (ASRC), Japan Atomic Energy Agency (JAEA), Tokai-mura, Ibaraki 319-1195 Japan
[2]Materials Sciences Research Centre (MSRC), Japan Atomic Energy Agency (JAEA), Tokai-mura, Ibaraki 319-1195 Japan
[3]Centre for Computational Science and e-Systems, JAEA, Kashiwa, Chiba 277-0871, Japan
[4]Nuclear Professional School, School of Engineering, The University of Tokyo, Ibaraki 319-1188 Japan
[5]Graduate School of Science and Engineering, Yamagata University, Yonezawa, Yamagata 992-8510, Japan
[6]National Institutes for Quantum Science and Technology, Takasaki-shi, Gunma 370-12929, Japan
[7]Nuclear Science and Engineering Centre (NSEC), Japan Atomic Energy Agency (JAEA), Tokai-mura, Ibaraki 319-1195 Japan

Author Note

*E-mail: aoyagi.noboru@jaea.go.jp

TABLE OF CONTENTS

1. **Supplementary Note 1: A set of Ce^{IV} concentration parameters for nanoclusters and ions obtained from SAXS analysis** **...3**

2. **Supplementary Note 2: The determination of the aggregation number (n_A) in a single secondary cluster** **...7**

3. **Supplementary Note 3: More details on the Raman spectroscopy result** **...9**

4. **Supplementary Note 4: CM-SANS datum extracted and the corresponding datum reduction** **...10**

1. Supplementary Note 1: A set of Ce^{IV} concentration parameters for nanoclusters and ions obtained from SAXS analysis

The SAXS intensity $I(q)$ obeys the following relationship:

$$I(q=0) = \phi_s \cdot V_s \cdot (\rho_s - \rho_m)^2, \qquad \text{(S1)}$$

where ϕ_s [N.D.] is the volume fraction of the secondary clusters of our concern, V_s [cm^3] is the volume of a single secondary particle, ρ_s and ρ_m are the scattering length density [cm^{-2}], respectively. Here, the summation of the volume fraction is

$$\phi_w + \phi_s + \phi_m = 1 \qquad \text{(S2)}$$

with the volume fraction of water, ϕ_w, and of matrix, ϕ_m, respectively. The matrix includes all of the ionic species in the system: $Ce_m(OH)_n^{(4m-n)+}$. In the present study, we assume m=1 and n=4 as the representative parameters with the one having close formula to the density available for relevant species ρ_s given in Table S1. Remember that this leads to a discrimination to obtain a proper solution of the polynomial in the present case.

Supplementary Table S1. Chemical formula, the density of the compounds, and scattering line density in the literature: 1-6

Chemical formula	density, D_x [Mg/m^3]	S.L.D. [Å^{-2}]		S.L.D. [cm^{-2}]		Ref.No.
		Re	Im	Re	Im	
H_2O	0.998 (25°C)	9.52E-06	-3.17E-06	9.52E+10	-3.17E+10	
Ce (Metal)	6.77	4.61E-05	-8.23E-06	4.61E+11	-8.23E+10	
CeO_2	7.20	5.13E-05	7.21E-06	5.13E+11	7.21E+10	1
$Ce^{III}(OH)_3$	4.57	3.40E-05	4.15E-06	3.40E+11	4.15E+10	2
$Ce^{III}(NO_3)_3(H_2O)_6$	2.40	1.97E-05	9.99E-07	1.97E+11	9.99E+09	3
$[NH_4]_2[Ce^{IV}(NO_3)_6]$	2.49	2.05E-05	1.08E-06	2.05E+11	1.08E+10	4
$[Ce^{IV}_2(\mu_2\text{-}OH)_2(NO_3)_6(H_2O)_8]$	2.48	2.02E-05	1.08E-06	2.02E+11	1.08E+10	5
$[Ce_6(\mu_6\text{-}O)(\mu_3\text{-}OH)_8(NO_3)_8]$	3.85	2.93E-05	2.71E-06	2.93E+11	2.71E+10	6

The volume fraction of the second clusters, ϕ_s [cm^3], is

$$\phi_s := \frac{C_s M_s}{d_s} \quad \frac{[\text{mol}][\frac{\text{g}}{\text{mol}}]}{[\frac{\text{g}}{\text{cm}^3}]}, \qquad \text{(S3)}$$

Where C_s [mol] is the number of the secondary clusters, M_s [g/mol] is the formula weight of the primary particle, d_s [g/cm^3] is the density of the particle. In the similar manner, we have the following fractions with C_m [mol] is the number of Ce ions in the matrix medium M_m [g/mol] is the formula weight of the ionic species, d_m [g/cm^3] is the density of the ionic species. Each fraction defines

$$\frac{\phi_w := \left(10^3 - \frac{C_s M_s}{d_s} - \frac{C_m M_m}{d_m}\right)}{10^3}, \qquad \text{(S4)}$$

and

$$\phi_m := \frac{C_m M_m}{10^3 d_m}, \qquad \text{(S5)}$$

where, the eq. S3 with concentration [mol/L] description provides dimensionless fraction for $\mathrm{k = w, s,}$ or m:

$$\phi_i = \frac{C_k M_k}{10^3 d_k}. \qquad \text{(S6)}$$

In the meantime, the scattering length density ρ_m in the eq(S1) is the summation of that of water and the matrix by a factor of their volume fraction χ_w and χ_I, respectively. The actual matrix contains a variety of the ionic species; however, we select $Ce(OH)_4$ as a presumable species in a succinct model case with a density of D_x = 2.49 [g/cm^3] (Table S1). As long as we investigated, no data is available for the density of $Ce(OH)_4$. This might be due to an extensive hygroscopic nature or the reason the determination of the hydration number

and the subsequent structures is very difficult. Therefore, we used $D_x = 2.49$ [g/cm^3] that is the value for CAN, instead. Some possible density candidates are listed in Table S1, ranging from 7.20 for CeO_2 to 2.40 for $Ce^{III}(NO_3)_3(H_2O)_6$. In reality, the system of our interest has a complicated mixture of the multiple components—the optimal value for the present second cluster might exist in between.

Note that ρ_I represents a scattering length density for the corresponding ion: $Ce(OH)_4$.

$$\rho_m = \chi_w \rho_w + \chi_i \rho_i \qquad \text{(S6)}$$

with

$$\chi_w = \frac{10^3 - \frac{C_s M_s}{d_s} - \frac{C_i M_i}{d_i}}{10^3 - \frac{C_s M_s}{d_s}} \qquad \text{(S7)}$$

and

$$\chi_i = \frac{\frac{C_i M_i}{d_i}}{10^3 - \frac{C_s M_s}{d_s}} \qquad \text{(S8)}$$

Total concentration of Ce^{4+} [mol/L] ions preserves;

$$\frac{C_t}{10^3} = \frac{C_s}{10^3} + \frac{C_i}{10^3} \qquad \text{(S9)}$$

In the case of $C_{Ce^{IV}}$ = 0.05 [M], which is the second series in the Procedure and Apparatus section, we substitute $\frac{C_t}{10^3} =$ 0.05 [M] for the total concentration. With the eq (S7) and the eq (S8), the eq (S6) turns into

$$C_i = -\frac{d_i}{d_s}\frac{M_s}{M_i}\frac{\rho_w - \rho_m}{\rho_w - \rho_i} C_s + 10^3 \frac{d_i}{M_i}\frac{\rho_w - \rho_m}{\rho_w - \rho_i} \;. \quad \text{(S10)}$$

And thus, one obtains the following simple solvable problem with a third order polynomial of ρ_{m} by using parameters in Table S2 and erasing C_{i} and C_{s} from the eq (S10) in combination with the eq (S9):

$$\rho_{\mathrm{m}}^3+b_2\rho_{\mathrm{m}}^2+b_1\rho_{\mathrm{m}}+b_0=0 \qquad \text{(S11)}$$

Supplementary Table S2. Parameters used for the calculation.

Given variables	value	unit	remark
$I(q=0)$	231.77	cm^{-1}	Exp.
V_s	8.01E-11	cm^{-2}	Exp.
ρ_{w}	9.44E+10	cm^{-2}	Exp.
ρ_{s}	5.20E+11	cm^{-2}	Exp.
ρ_{i}	1.88E+11	cm^{-2}	Exp.
b_2	−1.135E+12		numerical
b_1	3.67E+23		numerical
b_0	−2.564E+34		numerical

The numerical solutions of the eq (S11) are complex numbers with the negligible imaginary parts. Two of the solutions give the negative numbers for C_{i}. Therefore, an effective solution is:

$$\rho_{\mathrm{m}}= 9.58\mathrm{E}{+}10 \ \mathrm{cm}^{-2} \qquad \text{(S12)}$$

Finally, the corresponding concentrations are

$$C_{\mathrm{i}} = 6.97\mathrm{E}{-}03 \ \mathrm{M}, \quad \text{(S13)}$$

and

$$C_{\mathrm{s}} = 4.30\mathrm{E}{-}02 \ \mathrm{M}. \quad \text{(S14)}$$

2. Supplementary Note 2: The determination of the aggregation number (n_A) in a single secondary cluster

The volume of the secondary cluster satisfies the following relation with that of the primary cluster (V_p), the association number (n), and a filling factor (ζ)

$$V_s = V_p \cdot n \cdot \zeta \ , \qquad \text{(S15)}$$

Where the maximum number allowed is $\zeta_{max} \sim 0.64$ for a hexagonal closest packing. In the present spherical approximation, $V_s = (\frac{3}{4})\pi R_s^3$ and $V_p = (\frac{3}{4})\pi R_p^3$ provide the mean aggregation number of the secondary clusters: $\overline{n}$=27.0. The following scheme is an updated scheme described in the Introduction section via the present discussion.

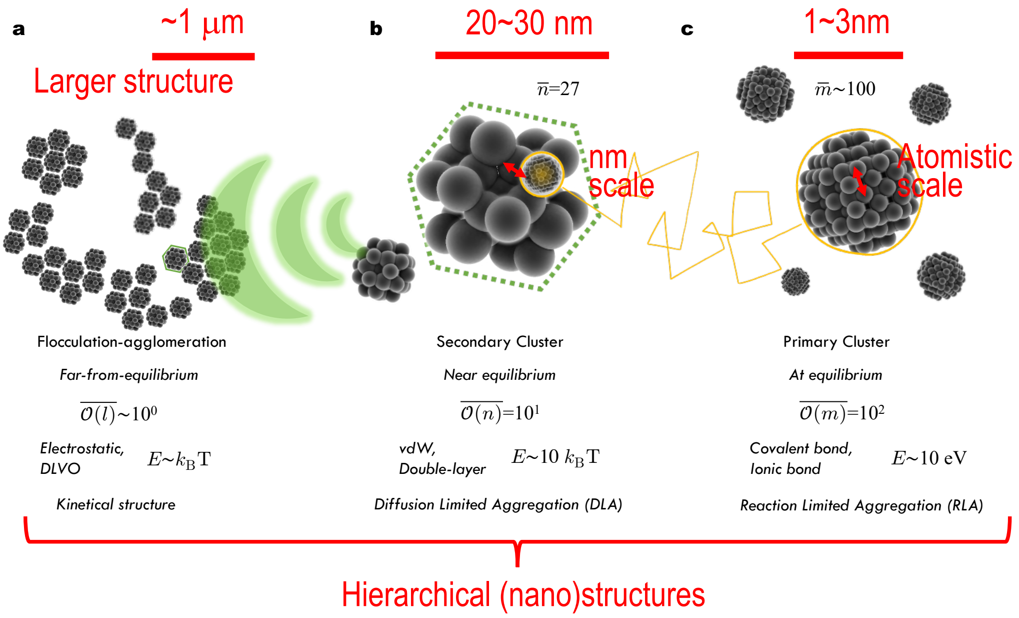

Supplementary Fig. S1. A hierarchical nanoarchitecture concluded in the present study. **a** Flocculation–agglomeration model. Common in kinetical structure observed on a carbon-coated copper microgrid. **b** Secondary cluster with the mean value of aggregation number $\overline{n}$ = 27. Diffusion Limited Aggregation is in control; that is, the Brownian motion (orange solid line segments represent a zigzag trace) is a key path. c Primary cluster with the mean value of Ce^{IV} in a single particle $\overline{m}$ ~ 100.

3. Supplementary Note 3: More details on the Raman spectroscopy result

Remarkably, the lower-energy band of nitrate displays multiple peaks (670 and 711 cm^{-1}) and is red-shifted for solutions at higher concentrations (spectra **b** and **c** in Fig. 4). The bound nitrate anion within pristine CAN favours bidentate coordination to one Ce^{IV} centre, with the vicinal hexa-nitrate sphere forming an icosahedral CeO_{12} core. The $[Ce(NO_3)_6]^{2+}$ has T_h (ideally O_h) molecular point group symmetry (shown in the monomer in Fig. 1). Notably, the Ce^{IV} complex commonly exhibits an isotropic structure. After dissolution in acid, the coordination mode of the nitrate to Ce^{IV} changes from bi- to monodentate due to primary cluster formation. Additionally, the nitrate is not bridging two Ce ions, and it is removed from the core with the "$–[O–Ce–O]–_n$" structure. Therefore, nitrate adsorption occurs on the core in a mono- or bidentate fashion, generating two peaks at 710 and 670 cm^{-1} that are red-shifted by ~40 and 80 cm^{-1} from 750 cm^{-1}, respectively. The bands at 1035, 750, and 252 cm^{-1} are assigned to NO_2 symmetric stretching and bending and Ce–O stretching vibrations, respectively.[7-10]

The secondary clusters exhibit a unique spectral peak at 451 cm^{-1} (indicated by the red "‡" in spectra **d** in Fig. 4), which has not been reported thus far, although non-negligible peaks have been recorded at approximately 606 cm^{-1}.[10] Based on a rough estimation of the particle size (58 nm), the band at 451 cm^{-1} is associated with phonon coupling (Table 1). In samples with higher $C_{Ce^{IV}}$ concentrations, this band was not observed due to excess ammonium cations, resulting in the absence of secondary clusters (spectra **b** and **c** in Fig. 4). Ce ion hydroxo-bridging causes the very weak, broad peaks at approximately 1340 and 1604 cm^{-1}.

However, these peaks are non-negligible and remarkable for primary clusters, indicating proton inclusion. The lowest-energy band at 98 cm^{-1} may be due to Ce–Ce interactions. However, these data are insufficient to identify either cluster type._

Meanwhile, we estimated the size of the single domain from the recorded Raman band width. We employed this relationship to determine the appropriate domain size in the liquid state, including the secondary cluster stability in a suspension. Weber *et al*. reported that particle-size effects influence the spectral broadening in Raman scattering,[11] and the half-width of the Raman band has the following linear relationship with the inverse crystalline size:

$$\Gamma = a + (b/D), \qquad (1)$$

where Γ (cm^{-1}) is the half-width at half-maximum of the observed Raman line, and D (nm) is the particle radius. Given the empirical constants of a = 5 cm^{-1} and b = 51.8 cm^{-1}/nm according to Weber *et al*., we obtained the particle sizes in Table 1 using this relationship. The obtained particle diameter (0.98 nm) is comparable to those obtained using small-angle X-ray scattering (SAXS) (1.64 nm, to be discussed later). However, this approach is only applicable to primary clusters because they form a crystalline lattice.

4. Supplementary Note 4: CM-SANS datum extracted and the corresponding datum reduction

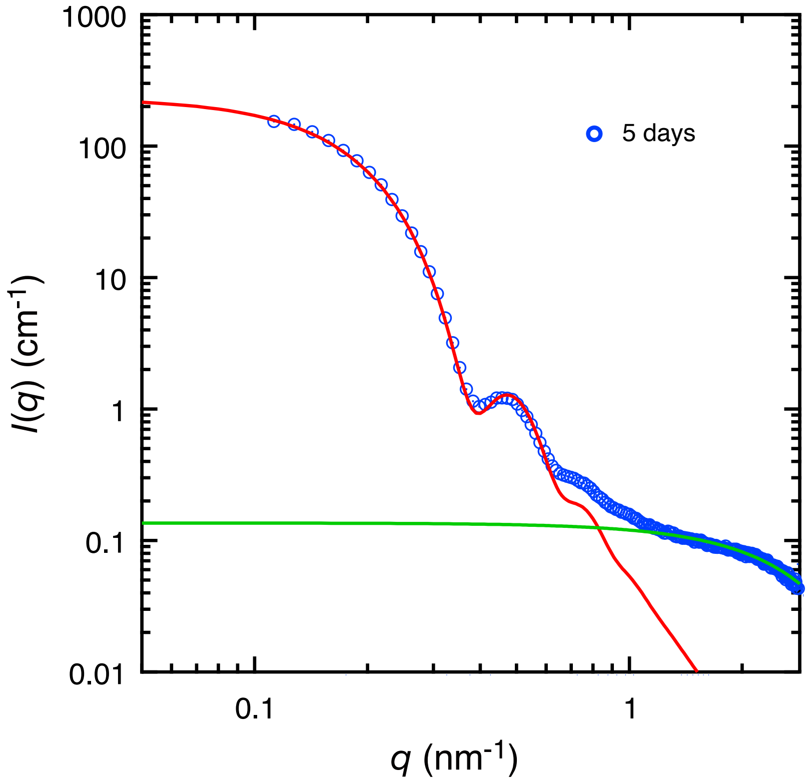

Supplementary Fig. S2. CM-SANS datum extracted and the corresponding datum reduction for clarity. The red and green curves represent the scattering contributions from the large R and small R structures, respectively.It is not easy to conclude the structural form of the fundamental unit etc; so it is prudent to state only the sizes.

Supplementary References

(1) Itoh, T.; Mori, M.; Inukai, M.; Nitani, H.; Yamamoto, T.; Miyanaga, T.; Igawa, N.; Kitamura, N.; Ishida, N.; Idemoto, Y. Effect of Annealing on Crystal and Local Structures of Doped Zirconia Using Experimental and Computational Methods. *J. Phys. Chem. C* **2015**, 119 (16), 8447-8458. DOI: 10.1021/jp5117118.

(2) Mullica, D. F.; Oliver, J. D.; Millgan, W. O. Cerium trihydroxide. *Acta Crystal. B* **1979**, 35 (11), 2668-2670. DOI: doi:10.1107/S0567740879010104.

(3) Milinski, N.; Ribar, B.; Sataric, M. Pentaaquatrinitratocerium(III) monohydrate, $Ce(H_2O)_5(NO_3)_3.H_2O$. *Cryst. Struct. Commun.* **1980**, 9 (2), 473-477.

(4) Beineke, T. A.; Delgaudio, J. Crystal structure of ceric ammonium nitrate. *Inorg. Chem.* **1968**, 7 (4), 715-721. DOI: 10.1021/ic50062a020.

(5) Guillou, N.; Auffrédic, J. P.; Louër, D. Synthesis, Crystal Structure, and Thermal Behavior of Cerium(IV) Oxide Nitrate $Ce_2O(NO_3)_6(H_2O)_6{\cdot}2H_2O$. *J. Solid State Chem.* **1994**, 112 (1), 45-52.DOI: https://doi.org/10.1006/jssc.1994.1262.

(6) Calvez, G.; Daiguebonne, C.; Guillou, O.; Le Dret, F. A New Series of Anhydrous Lanthanide-Based Octahedral Hexanuclear Complexes. *Eur. J. Inorg. Chem.* **2009**, (21), 3172-3178, https://doi.org/10.1002/ejic.200900283. DOI: https://doi.org/10.1002/ejic.200900283 (acccessed 2021/03/29).

(7) Miller, J. T.; Irish, D. E. Infrared and Raman spectra of the cerium(IV) ion – nitrate ion – water system. *Can. J. Chem.* **1967**, 45 (2), 147-155. DOI: 10.1139/v67-030 (acccessed 2019/06/28).

(8) Larsen, R. D.; Brown, G. H. The Structure of Ammonium Hexanitratocerate(IV) in Solution1a. *J. Phys. Chem*. **1964**, 68 (10), 3060-3062. DOI: 10.1021/j100792a502.

(9) Briois, V.; Lützenkirchen-Hecht, D.; Villain, F.; Fonda, E.; Belin, S.; Griesebock, B.; Frahm, R. Time-Resolved Study of the Oxidation of Ethanol by Cerium(IV) Using Combined Quick-XANES, UV−Vis, and Raman Spectroscopies. *J. Phys. Chem. A* **2005**, 109 (2), 320-329. DOI: 10.1021/jp046691t.

(10) Demars, T. J.; Bera, M. K.; Seifert, S.; Antonio, M. R.; Ellis, R. J. Revisiting the Solution Structure of Ceric Ammonium Nitrate. *Angew. Chem. Int. Ed.* **2015**, 54 (26), 7534-7538. DOI: 10.1002/anie.201502336.

(11) Weber, W. H.; Hass, K. C.; McBride, J. R. Raman study of CeO_2 Second-order scattering, lattice dynamics, and particle-size effects. *Phys. Rev. B* **1993**, 48 (1), 178-185. DOI: 10.1103/Phys. Rev. B.48.178.
